# Professionals’ knowledge, skills and confidence on using the best practices for spinal cord injury physical activity counseling in Canada and the Netherlands

**DOI:** 10.1080/10790268.2024.2391595

**Published:** 2024-09-11

**Authors:** Laura Kuipers, Trynke Hoekstra, SCI Physical Activity Counseling Panel, Femke Hoekstra

**Affiliations:** 1 Department of Health Sciences and Amsterdam Public Health research institute, Vrije Universiteit Amsterdam, Amsterdam, Netherlands; 2 University of British Columbia, Kelowna, Canada; 3 School of Health and Exercise Sciences, University of British Columbia, Kelowna, Canada; 4 International collaboration on Repair Discoveries (ICORD), University of British Columbia, Vancouver, Canada

**Keywords:** Spinal cord injury, Physical activity, Counseling

## Abstract

**Context:**

To improve physical activity (PA) participation in people with spinal cord injury (SCI), an international panel co-created theory- and evidence-based best practices for SCI PA counseling. This study aimed to identify and compare Canadian and Dutch counselors’ knowledge, skills, and confidence in using these best practices.

**Methods:**

An online survey was conducted in Canada and the Netherlands. Respondents were included if they worked or volunteered as exercise/lifestyle counselor, recreation therapist, physiotherapist, occupational therapist, or peer mentor and were planning to provide counseling in the next 12 months. Chi-square tests, t-tests and linear regression analyses were used to compare groups.

**Results:**

Canadian (*n* = 45) and Dutch respondents (*n* = 41) had different expertise, with the majority of Canadians working as therapeutic recreation therapist and the majority of Dutch respondents working as PA/lifestyle counselor. In both countries, respondents scored relatively high on their knowledge, skills, and confidence in using the best practices on how to have a conversation and what to discuss during a conversation. Dutch respondents scored slightly higher in their confidence for using best practices about building rapport, motivational interviewing, and tailoring the support (*p* = 0.05).

**Conclusions:**

The generally high counseling skills reported by Canadian and Dutch respondents may be due to the history of SCI-specific PA promotion projects conducted in both countries. These survey findings were used to inform the development of evidence-based training modules on SCI PA counseling. This study may inspire cross-country collaboration and exchange to optimize the organization and delivery of PA counseling services for adults with SCI.

## Introduction

Many people with spinal cord injury (SCI) are not sufficiently active to achieve fitness and health benefits (1,2). Providing physical activity (PA) counseling support^1^ can increase PA participation and improve health and well-being in people with SCI (3–6). A systematic review on the effectiveness of behavior change techniques (BCTs) in SCI PA self-management interventions reported 222 different BCTs from 31 studies with mixed effectiveness (7). These and other findings (8,9) illustrate the diversity of PA counseling support deliverance. Furthermore, counseling is given in different settings (e.g. rehabilitation, peer mentorship programs) by counselors with different expertise (e.g. physiotherapists, occupational therapists, personal trainers, peer mentors) (10–12). This variety may suggest that counselors have different knowledge and skills for providing PA counseling (13), which may explain the variability in SCI PA counseling efforts (14) and clients’ behavior outcomes.

**Figure 1:**
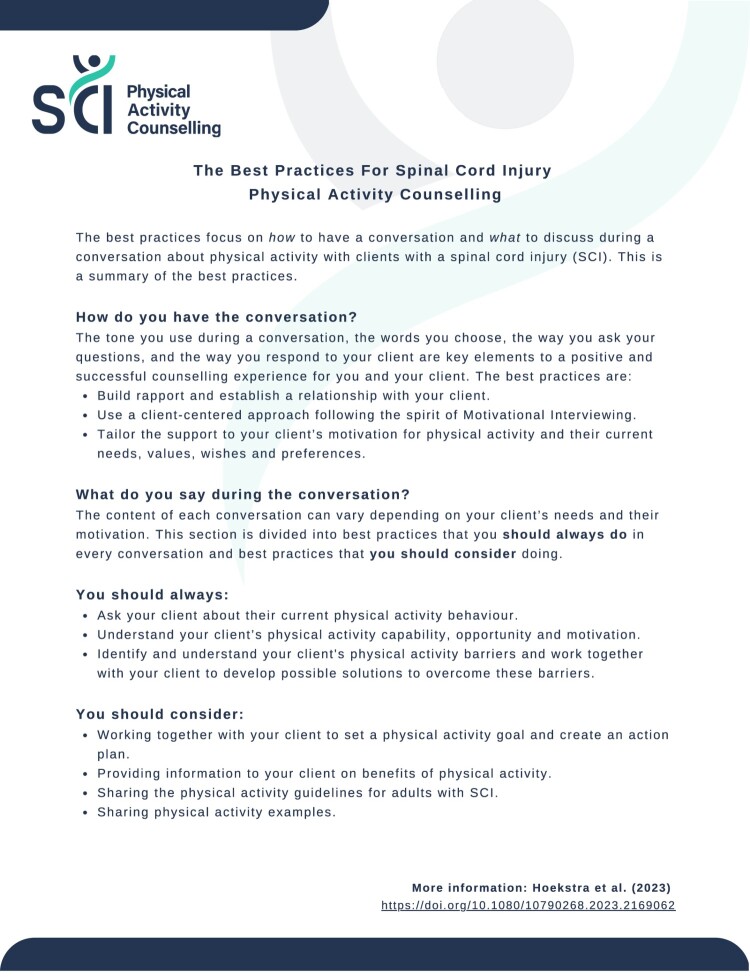
A summary of the best practices for spinal cord injury physical activity counseling. Figure adapted from Hoekstra *et al*. (13). Theory- and evidence-based best practices for physical activity counseling for adults with spinal cord injury. The journal of spinal cord medicine, 1–13 (13).

Access to evidence-based resources and training may contribute to increasing counselors’ knowledge and skills on SCI PA counseling and, subsequently, contribute to improving the support they provide to clients with SCI (15). Recently, an international multidisciplinary expert panel published theory  – and evidence-based best practices for SCI PA counseling (13). The best practices were rigorously developed and informed by behavior change theory, SCI PA counseling literature, and panel members’ knowledge and experiences. The best practices entail best practices on *how* to have a conversation and best practices on *what* to discuss about PA (Figure 1) (13). The Panel recommended developing training tools to teach counselors these best practices (13). To tailor the development of these tools to the context in which they will be implemented (16,17), insights are needed into counselors’ current knowledge, skills, confidence, barriers, and needs regarding providing and improving SCI PA counseling support.

Canada and the Netherlands can be considered as leading countries in the development, evaluation, and implementation of PA counseling programs for people with SCI. A logical first step is to start the implementation of the best practices in these countries. However, different approaches may be needed in Canada and the Netherlands due to differences in infrastructure, and existing PA promotion policies and programs (18). Understanding and comparing Canadian and Dutch counselors’ knowledge, skills, and confidence in using the SCI PA counseling best practices can provide new insights to optimize the organization and delivery of SCI PA counseling services within and beyond Canada and the Netherlands. This study aimed to (1) identify and compare Canadian and Dutch counselors’ knowledge, skills, and confidence related to using the SCI PA counseling best practices and (2) identify counselors’ barriers and needs to improve SCI PA counseling, with the ultimate aim to inform the development of the training modules.

## Methods

The online survey was developed and disseminated in Canada and the Netherlands, in partnership with the SCI Physical Activity Counseling Panel (Supplementary A). The Integrated Knowledge Translation (IKT) guiding principles for conducting SCI research in partnership were used to guide the collaborative activities (19,20). The study was approved by the UBC Okanagan Behavioral Research Ethics Board.

### Study population

Respondents were included if they work or volunteer as an exercise/lifestyle counselor, occupational therapist, recreation therapist, psychomotor therapist, physiotherapist, kinesiologist, rehabilitation assistant, social worker, fitness trainer or coach, SCI peer mentor, or SCI caregiver in Canada or the Netherlands; and are over the age of 18 and planning to provide PA counseling to one or more adults in the next 12 months.

### Data collection

Recruitment strategies are described in Supplementary file B. We used a variety of recruitment strategies. First, we asked members of the SCI Physical Activity Counseling Panel to share this study opportunity among their network. Second, we reached out to >45 national and provincial organizations representing various counseling professions to promote the study among their members. The survey included questions on demographic information, counseling experience, knowledge, skills, needs, and barriers to applying the best practices for SCI PA counseling (Supplementary C for an overview of the survey variables). Questions on knowledge and skills were about physical capability to use a best practice, while questions on confidence levels were about psychological capability (21). Each of these questions had answer options according to a 7-point Likert scale.

The survey was informed by SCI PA counseling best practices (13), preexisting surveys from PA promotion studies (22–24), and behavior change theory (21,25). The SCI PA Counseling Panel had opportunities to provide feedback on survey questions. The English survey was translated into Dutch by a professional translator and checked by Dutch team members (FH, LK). The English survey was disseminated in March 2022, and the Dutch version in April 2022. Data were collected until June 2022.

### Data analysis and statistics

Statistical analyses were conducted using a complete case sample. Missing data were analyzed in a sensitivity analysis by comparing complete cases with incomplete cases (Supplementary D). Fisher’s exact or Chi-square tests (for comparison of categorical variables) and t-test (for comparison of continuous variables) were conducted to compare differences between the Netherlands and Canada (see Tables 1–3). Linear regression analysis was performed with sum scores of the best practices on *how* to have the conversation and *what* to discuss, separately for knowledge and skills and confidence levels (see Supplementary file E, Table 3). A significance level with a *P*-value of 0.05 was used and analyses were conducted using IBM SPSS Statistics version 25.

**Table 1:** Study population demographic characteristics of Canadian and Dutch respondents.

	Canada (*n* = 45)	The Netherlands (*n* = 41)	*P*-value
Mean age in years ± SD (range)	35 ± 12 (21–62)	39 ± 11 (24–69)	0.09
Gender identity (%):			0.23
Woman	36 (80.0)	32 (78.0)	
Man	6 (13.3)	9 (22.0)	
Genderqueer/gender non-conform	0 (0)	0 (0)	
Non-binary	0 (0)	0 (0)	
Transgender man	0 (0)	0 (0)	
Transgender woman	0 (0)	0 (0)	
Different identity	0 (0)	0 (0)	
Prefer not to answer	3 (6.7)	0 (0)	
Identify as a person with a SCI	5 (11.1)	2 (4.9)	0.44
Has ever spent 24 consecutive hours with an individual with a SCI	10 (22.2)	10 (24.4)	0.81
Background and expertise (%)*:			
Exercise or lifestyle counselor	14 (31.1)	20 (48.8)	0.09
Therapeutic recreation therapist	19 (42.2)	3 (7.3)	<**0.005**
SCI peer mentor	6 (13.3)	2 (4.9)	0.27
Physiotherapist	5 (11.1)	11 (26.8)	0.06
Occupational therapist	4 (8.9)	5 (12.2)	0.73
Other	19 (42.2)	12 (29.3)	
Professional background/role (%):			**<0**.**005**
Employed full-time	26 (57.8)	30 (73.2)	
Employed part-time	5 (11.1)	9 (22.0)	
Unemployed and looking for work	1 (2.2)	0 (0)	
Unemployed and not looking for work	0 (0)	0 (0)	
Self-employed	1 (2.2)	1 (2.4)	
Retired	1 (2.2)	1 (2.4)	
Unable to work	1 (2.2)	0 (0)	
Student	10 (22.2)	0 (0)	
How many adults with SCI do you counsel per year? (%)			**0**.**02**
0	2 (4.4)	9 (22.0)	
1–4	26 (57.8)	16 (39.0)	
5–9	3 (6.7)	2 (4.9)	
10–14	5 (11.1)	0 (0)	
15–24	2 (4.4)	3 (7.3)	
>25	7 (15.6)	11 (26.8)	
How many years of experience do you have in providing physical activity counseling?			0.98
None	2 (4.4)	3 (7.3)	
Less than 1 year	8 (17.8)	7 (17.1)	
Between 1–3 years	10 (22.2)	9 (22.0)	
More than 3 years	25 (55.6)	22 (53.7)	
Has received a formal training or workshop in Motivational Interviewing	23 (51.1)	31 (75.6)	**0**.**02**
Has received other formal training or workshops in behavior change or counseling techniques	31 (68.9)	17 (41.5)	**0**.**01**

*Notes*: Age was calculated by the formula “current year minus year of birth.” *Respondents could select multiple background/expertise categories they belong in, therefore the *P*-value for the difference between Canadian and Dutch respondents is calculated for each category. No *P*-value is calculated for the category “other” because groups that fall under this category differ. Expertise groups that fell under the category “exercise or lifestyle counselor” included the Dutch expertise groups “buurtsportcoach” (Neighborhood Sports Coach) and “bewegingsagoog.” The Dutch expertise group “activiteitenbegeleider” fell under “therapeutic recreation professional.” Groups that fell under the category “other” were: fitness trainer, kinesiologist, researcher, medical specialist, nurse specialist, rehabilitation assistant, psychomotor therapist, community organization representative, and social worker.

**Table 2:** Knowledge of physical activity guidelines of Canadian and Dutch respondents.

	Canada (*n* = 45)	The Netherlands (*n* = 41)	*P*-value
**Physical activity guidelines (%)**	** **	** **	** **
Do you know the SCI-specific physical activity guidelines? (%)			0.87
Yes	17 (37.8)	15 (36.6)	
No, but I have heard of these guidelines	16 (35.6)	13 (31.7)	
No, I do not know and have not heard of these guidelines	12 (26.7)	13 (31.7)	
Used the SCI-specific physical activity guidelines (%)*	13 (76.5)	11 (73.3)	1.00
Selected the right current recommended Canadian/Dutch physical activity guidelines for improving fitness in adults with SCI (%)*	13 (76.5)	7 (46.7)	0.08
Selected the right current World Health Organization’s Recommended physical activity guidelines for adults aged 18 years and older (%)	18 (40.0)	12 (29.3)	0.16

*Notes*: *These questions were only asked to respondents that selected “Yes” on the question “Do you know the SCI-specific physical activity guidelines?”

**Table 3:** Barriers and needs on improving SCI PA counseling of Canadian and Dutch respondents.

	Canada (*n* = 45)	The Netherlands (*n* = 41)	*P*-value
Are you experiencing any barriers to improving your knowledge and skills on SCI-specific physical activity counseling?* (%)			
No, I do not experience any barriers	2 (4.4)	8 (19.5)	**0.04**
Lack of time	33 (73.3)	22 (53.7)	0.06
Lack of reimbursement	22 (48.9)	8 (19.5)	**<0**.**005**
Lack of resources	11 (24.4)	4 (9.8)	0.07
Lack of interest	2 (4.4)	2 (4.9)	1.00
Feeling it would not be beneficial to me	2 (4.4)	0 (0)	0.50
Disbelief that it will change my counseling knowledge/skills	0 (0)	1 (2.4)	0.48
Other^a^	8 (17.8)	13 (31.7)	
Are you experiencing any barriers to improving your confidence in delivering physical activity counseling to adults with SCI?* (%)			
No, I do not experience any barriers	8 (17.8)	17 (41.5)	**0**.**02**
Lack of practice	27 (60.0)	14 (34.1)	**0**.**02**
Lack of knowledge	23 (51.1)	13 (31.7)	0.07
Lack of skills	13 (28.9)	7 (17.1)	0.20
Feeling it would not be beneficial to me	1 (2.2)	1 (2.4)	1.00
Disbelief that it will change my confidence level	1 (2.2)	1 (2.4)	1.00
Other^b^	5 (11.1)	3 (7.3)	
I want to improve my knowledge and skills on SCI-specific physical activity counseling°			
Mean ± SD (range)	6.3 ± 0.7 (5–7)	5.2 ± 1.4 (1–7)	**<0**.**005**
Would use an online resource/toolkit outlining the evidence-based			
best practices (%)	45 (100.0)	35 (85.4)	**0**.**01**
Would be interested in online training modules that are free of charge to teach counselors the best practices (%)	45 (100.0)	36 (87.8)	**0**.**02**

*Notes*: *Respondents could select multiple barriers, therefore the *P*-value for the difference between Canadian and Dutch respondents is calculated for each category. No *P*-value is calculated for the category “other” because groups that fell under this category differ. ^a^For both countries, most other barriers were not being familiar or working with a few SCI clients. Furthermore, not knowing what resources are available and practical barriers such as travel costs for trainings, lack of facilities, and closure of PA centers were mentioned. ^b^A respondent in both countries mentioned improving their confidence was dependent on the client counseled, and how often SCI counseling was done. Other barriers mentioned for Canada were (lack of) opportunities to discuss with others and find resources. For the Netherlands, it was mentioned there should be more practical training. °Measured on a 7-point Likert scale (1 = strongly disagree, 2 = disagree, 3 = somewhat disagree, 4 = neither agree nor disagree, 5 = somewhat agree, 6 = agree, 7 = strongly agree).

## Results

We enrolled 45 Canadian and 41 Dutch respondents. The mean age of Canadian and Dutch respondents was 35 years (SD = 12) and 39 years (SD = 11), respectively (Table 1). In both countries, respondents predominantly identified themselves as a woman (Canada 80%; Netherlands 78%). Some respondents identified as a person with SCI: 11% of Canadian respondents and 5% of Dutch respondents. More than a fifth (22%) of Canadians and almost 25% of Dutch respondents had spent 24 consecutive hours with someone with SCI.

The majority of Canadian respondents worked as recreation therapist (42%) and/or PA/lifestyle counselor (31%). The majority of Dutch respondents worked as PA/lifestyle counselor (49%) and/or physiotherapist (27%). Of the Canadian respondents, 58% supported 1–4 clients with SCI per year, compared to 39% of Dutch respondents. Just over a fifth of Dutch respondents had no clients with SCI in an average work year (22%), which was substantially more than in the Canadian group (4%). More Dutch respondents had received formal training in Motivational Interviewing (MI) compared to Canadians (Canada 51%; Netherlands 76%).

### Knowledge and skills

**Figure 2:**
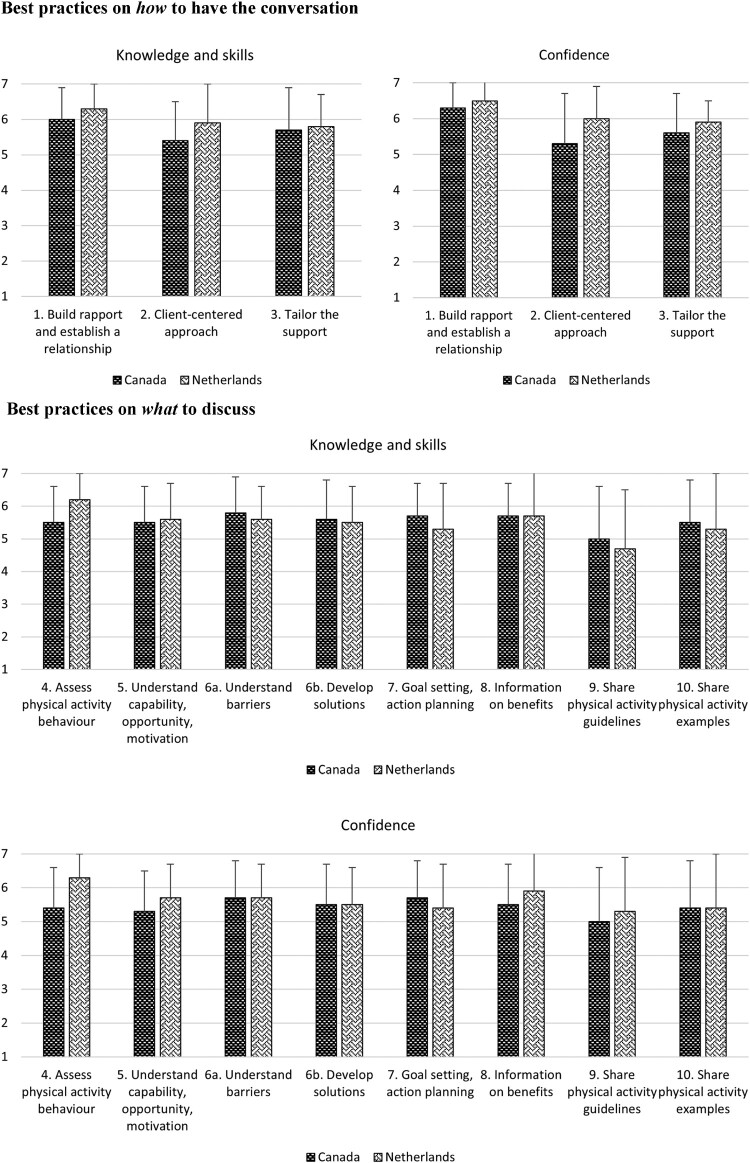
Knowledge, skills, and confidence to use the best practices. Rated on a 7-point Likert scale (1 = strongly disagree, 2 = disagree, 3 = somewhat disagree, 4 = neither agree nor disagree, 5 = somewhat agree, 6 = agree, 7 = strongly agree). See Supplementary E Table 2 for the mode and median, in the graphs the mean and SD are shown (excluding respondents that selected “unsure”) for easy interpretation of the results.

Canadian and Dutch respondents scored generally high on their knowledge, skills and confidence levels for using the SCI PA counseling best practices (Figure 2). Regarding confidence levels on how to have a PA conversation, Dutch respondents scored themselves generally higher compared to Canadians. These best practices focused on building rapport, using a client-centered approach in the spirit of MI and tailoring the support. The mean difference was 0.94 points higher (95% CI 0.01–1.86) for Dutch respondents compared to Canadians (Supplementary E). Regarding the best practices on what to discuss during a PA conversation, Dutch respondents tend to score lower in knowledge and skills but higher in confidence to implement these best practices. However, these results were not statistically significant (Supplementary E).

Considering the SCI PA guidelines, 38% of Canadian and 37% of Dutch respondents selected that they knew the guidelines (Table 2). Around three-quarters of those respondents used them (Canada 77%; Netherlands 73%). However, less than half of Dutch respondents selected the right SCI PA guidelines in a multiple-choice question (Netherlands 47%).

Most respondents experienced barriers to improving their knowledge and skills, with only 4% of Canadian respondents experiencing no barriers compared to 20% of Dutch respondents (Table 3). Almost half of Canadian respondents reported a lack of reimbursement as a barrier (49%) compared to a fifth of Dutch respondents (20%). Lack of resources was also a barrier. Especially many Dutch respondents reported no barriers to improving their confidence (42%). Among those that did report barriers to improving their confidence, lack of practice (Canada 60%; Netherlands 34%) and lack of knowledge (Canada 51%; Netherlands 32%) were frequently selected barriers.

Both Canadian and Dutch respondents (somewhat) agreed they wanted to improve their knowledge and skills, and all Canadian (100%) and most Dutch respondents (85%) would use online training outlining the best practices.

## Discussion

This study showed that, in general, Canadian and Dutch survey respondents were experienced and knowledgeable about delivering PA counseling to adults with SCI. Compared to Canadians, Dutch respondents scored slightly higher in their confidence in how to have a PA conversation with their clients with SCI. Still, counselors wanted to improve their knowledge and skills and experienced barriers to doing so. Study findings were used to inform the development of training modules for SCI PA counseling to further optimize SCI PA counseling services within and beyond Canada and the Netherlands.

The generally high scores of counselors’ knowledge, skills, and confidence found in this study may be explained by the many SCI-specific PA-promoting activities developed, studied, and implemented in Canada (9,11,26) and the Netherlands (9). In Canada, a variety of community-based PA programs exist for people with SCI, including tele-health programs, peer-delivered and group-based programs (11). In the Netherlands, SCI counseling support is structurally integrated into rehabilitation care via PA counseling centers (27). We may have reached mainly survey respondents involved in one of these support programs explaining the high knowledge, skills, and confidence due to their counseling training and experiences. Despite these high levels, respondents still wanted to improve their knowledge and skills in SCI PA counseling.

We identified three key differences between Canadian and Dutch respondents. First, Dutch respondents rated their confidence in using the best practices slightly higher than Canadians. A possible explanation could be that Dutch respondents received more extensive MI training. MI is widely accepted as a gold standard for delivering any type of behavioral support in the Netherlands (28) and integrated into educational programs for lifestyle counselors, occupational therapy, physiotherapy (28–31), and initiatives for evidence-based behavior change in healthcare (27,32–36).

Second, Canadians seemed to report more barriers to improving their knowledge, skills, and confidence. This may be due to cultural and infrastructural differences. There are relatively high standards of performance in work in Canada (37,38), while in the Netherlands consensus and solidarity are considered important (37,39,40). Lack of resources and reimbursement were more frequently reported in Canada, which may be due to differences in population density between both countries (41,42). Barriers of lack of time and resources may be caused by challenges in organizing care for people with SCI in large countries like Canada. Intersectoral collaboration and transfers may be easier to manage in a small country like the Netherlands, which has specific jobs for intersectoral collaboration (e.g. Neighborhood Sports Coaches) (43).

Third, Canadian respondents selected more often the correct recommendations for the SCI PA Guidelines compared to Dutch respondents. A possible explanation may be that the SCI Fitness Guidelines were first developed and disseminated in 2011 by a Canadian-led research group (44). While these fitness guidelines were translated into Dutch, active promotion and dissemination of the SCI exercise guidelines started after the updated scientific international SCI exercise guidelines were developed in 2018 (44).

This study has some limitations. First, no validated survey was used because the study aimed to explore knowledge, skills, and confidence levels of counselors for the newly developed best practices. The survey was based on theory and preexisting surveys and developed in collaboration with the SCI Physical Activity Counseling Panel. Second, a broad definition of PA counseling was used and this study had a relatively small sample size. Due to the descriptive nature of our findings, results should be approached with caution. However, despite the small sample size, our sample did exhibit diversity in terms of experience, and cultural backgrounds. The descriptive findings of this study have been used to tailor the development of training modules on SCI PA counseling to professionals’ challenges to improving their knowledge, skills, and confidence levels (45). More research is needed to identify the needs of counselors with other expertise and those who are less experienced. Furthermore, most respondents identified themselves as a woman, were straight, and of European descent. To better understand health disparities, people from different ethnicity, gender, and sexual orientations should be included in future studies.

## Conclusions

Although the counselors generally reported high counseling skills, they were interested in improving their knowledge and skills by participating in a training module. These descriptive findings were used to inform the development of evidence-based training modules on SCI PA counseling. This study may inspire cross-country collaboration and exchange to optimize the organization and delivery of PA counseling services for adults with SCI.

## Supplementary Material

Supplemental Material

## Footnotes

1 Physical activity counseling is defined as “any type of behavioral support (conversation) on starting, changing, and/or maintaining a physically active lifestyle between a counselor or individual and a client” (13).
