# Supplementary material for: Professionals’ knowledge, skills and confidence on using the best practices for spinal cord injury physical activity counseling in Canada and the Netherlands
Source: J Spinal Cord Med. 2024 Sep 11;48(1):148–57. doi: 10.1080/10790268.2024.2391595 (PMC11760748; doi:10.1080/10790268.2024.2391595)
Supplement: Supplemental Material [file YSCM_A_2391595_SM0314.docx]

**Supplementary files**

**Table of Contents**

**Supplementary A:** SCI Physical Activity Counseling Panel

**Supplementary B:** Recruitment strategies

**Supplementary C:** Overview of the survey variables

**Supplementary D:** Results with incomplete cases included

**Supplementary E:**  Results

**References**

**Open Science Framework:**

- Consent forms (Dutch + English version)

- Survey (Dutch + English version)

*Link to the Open Science Framework page:*

<https://osf.io/hj5ys/>

**Supplementary A. SCI Physical Activity Counseling Panel** (1)

**Table 1 SCI Physical Activity Counseling Panel members** panel members’ names, expertise, background and hierarchical relationships between panel members (1)

| Names | Organization | Country | Expertise / background |
| --- | --- | --- | --- |
| Anniek van Vilsteren | Vogellanden | NL | Counsellor + Psychomotor therapist |
| Delaney Collins | UBC / Dalhousie University | Canada | Counsellor + Occupational therapy student |
| Diane Rakiecki | UBC | Canada | Physical education (BPE) background + lived experience in SCI |
| Emily Giroux | UBC | Canada | Counsellor + trainer + researcher + partner of someone with SCI |
| Erica de Passillé | Horizon Health Network | Canada | Physiotherapist |
| Femke Hoekstra | UBC | Canada & NL | Researcher |
| Heather Gainforth | UBC | Canada | Researcher |
| Jasmin Ma | UBC | Canada | Researcher + kinesiologist + counsellor + trainer |
| Jereme Wilroy | University of Alabama at Birmingham | USA | Health coach + lived experience in SCI + researcher |
| Kathleen Martin Ginis | UBC | Canada | Researcher |
| Megan Williamson | Ocean Rehab and Fitness | Canada | Adaptive fitness coach + educator |
| Rita van den Berg-Emons | Erasmus MC | NL | Researcher |
| Rogier Broeksteeg | Rijndam Revalidatie | NL | Counsellor + health coach + physiotherapist |
| Shannon McCallum | Therapeutic Recreation Program | Canada | Recreation therapist + professor |
| Shannon Rockall | Praxis Spinal Cord Institute | Canada | Occupational therapist |
| Sonja Gaudet | UBC + SCI BC | Canada | SCI peer mentor + counsellor + lived experience in SCI |
| Stephanie Corras | Get in Motion coordinator | Canada | Counselling services + training counsellors |

Further information on panel selection, considerations and group-level demographics are described in Hoekstra et al. (2023) (1).

.

**Supplementary B. Recruitment strategies**

Various strategies were used to recruit respondents. Members of the SCI Physical Activity Counseling Panel were asked to distribute the survey among their networks. For example, the SCI Action Canada Lab had strong relationships with community organizations and peer mentors (2), and rehabilitation professionals of Dutch PA programs for people with a disability were approached, ReSpAct and Stichting Special Heroes (3, 4). An overview of Canadian SCI community-based organizations was used (5). For both countries, rehabilitation centers, hospitals and national and provincial associations of counselors were approached, such as associations of physiotherapists, occupational therapists and therapeutic recreation therapists. Respondents were approached via email and social media platforms Twitter, Facebook and LinkedIn.

A gift card of 10 Canadian dollars or 7 euro was given after completing the survey to compensate respondents for their time.

**Supplementary C. Overview of the survey variables**

See Open Science framework for the Dutch and English Survey.

**Table 1 Demographic information and counseling experiences** Multiple choice and dichotomous (Yes/No) answer options.

| Construct | Options | Reference |
| --- | --- | --- |
| Demographics |  |  |
| Age | Age of birth dropdown menu | - |
| Province/territory in Canada | Multiple choice | (6) |
| Gender identity | Multiple choice | (7) |
| Sexual orientation | Multiple choice | (8) |
| Ethnicity | Canadian survey: multiple choice (multiple answers possible)  Dutch survey: multiple choice | (9) |
| Identify as person with spinal cord injury | Yes/No | (10) |
| Spent 24 hours with an individual with spinal cord injury | Yes/No | - |
| Counseling experiences |  |  |
| Expertise/background | Multiple choice (multiple answers possible) | (1) |
| Professional background or role | Multiple choice | (1) |
| Amount of clients with spinal cord injury per year | Multiple choice | - |
| Years of experience in providing physical activity counseling | Multiple choice | - |
| Received formal training/workshop in Motivational Interviewing | Yes/No | (11, 12) |
| Received other formal training/workshops in behavioral change or counseling techniques | Yes/No | (1, 12) |

**Table 2 Knowledge and skills** Multiple choice, dichotomous (Yes/No) and 7-point Likert scale answer options. Knowledge and skills were measured with statements of the best practices for SCI PA counseling.

| Construct |  | Answer options | Theory/approach | Reference |
| --- | --- | --- | --- | --- |
| Know SCI-specific PA guidelines |  | Multiple choice | Physical activity guidelines | (13) |
| Current recommended Canadian SCI-specific PA guidelines |  | Multiple choice | Physical activity guidelines | (13) |
| Current recommended WHO PA guidelines |  | Multiple choice | Physical activity guidelines | (14) |
| Used SCI-specific PA guidelines for exercise counseling |  | Yes/No | Physical activity guidelines | (13) |
| Knowledge and skills | **Confidence in** |  |  |  |
| to build rapport and establish a relationship | ability to build rapport and establish a relationship | 7-point Likert scale (Agree-disagree) and ‘Unsure’ option | COM-B (capability), MI | (1, 15) |
| to use a client-centered approach following the spirit of Motivational Interviewing | ability to use a client-centered approach following the spirit of Motivational Interviewing | 7-point Likert scale (Agree-disagree) and ‘Unsure’ option | COM-B (capability), MI | (1, 15) |
| to tailor the support to motivation for physical activity and current needs, values, wishes and preferences | ability to tailor the support to motivation for physical activity and current needs, values, wishes and preferences | 7-point Likert scale (Agree-disagree) and ‘Unsure’ option | COM-B (capability, motivation), MI | (1, 15) |
| to assess current physical activity behavior | ability to assess current physical activity behavior | 7-point Likert scale (Agree-disagree) and ‘Unsure’ option | COM-B (capability) | (1, 15) |
| to understand physical activity capability, opportunity and motivation | ability to understand physical activity capability, opportunity and motivation | 7-point Likert scale (Agree-disagree) and ‘Unsure’ option | COM-B (capability, opportunity, motivation) | (1, 15–18) |
| to identify and understand physical activity barriers | ability to identify and understand physical activity barriers | 7-point Likert scale (Agree-disagree) and ‘Unsure’ option | COM-B (capability, opportunity, motivation) | (1, 15–18) |
| to work together to develop possible solutions to overcome physical activity barriers | ability to work together to develop possible solutions to overcome physical activity barriers | 7-point Likert scale (Agree-disagree) and ‘Unsure’ option | COM-B (capability, opportunity, motivation) | (1, 15–18) |
| to work together to set a physical activity goal and create an action plan | ability to work together to set a physical activity goal and create an action plan | 7-point Likert scale (Agree-disagree) and ‘Unsure’ option | COM-B (capability) behavior change techniques (goal setting, action planning) | (1, 15, 19, 20) |
| to provide information on benefits of physical activity | ability to provide information on benefits of physical activity | 7-point Likert scale (Agree-disagree) and ‘Unsure’ option | COM-B (capability) | (1, 15, 17, 21, 22) |
| to share the SCI-specific physical activity guidelines | ability to share the SCI-specific physical activity guidelines | 7-point Likert scale (Agree-disagree) and ‘Unsure’ option | COM-B (capability), physical activity guidelines | (1, 13, 15) |
| to share physical activity examples | ability to share physical activity examples | 7-point Likert scale (Agree-disagree) and ‘Unsure’ option | COM-B (capability) | (1, 15, 23–25) |

**Table 3 Barriers** Response options multiple choice or according to a 7-point Likert scale.

| Construct | Options | Theory/approach | Reference |
| --- | --- | --- | --- |
| Barriers |  |  |  |
| Barriers to improving knowledge and skills | Multiple choice (multiple answers possible): ‘No barriers’, ‘Lack of time’, ‘Lack of reimbursement’, ‘Disbelief that it will change my knowledge/skills’, ‘Lack of resources’, ‘Lack of interest’, ‘Feeling it would be beneficial for me’, ‘Other, namely [text entry]’ | COM-B (capability, opportunity, motivation) | (12, 15, 26, 27) |
| Barriers to improving confidence | Multiple choice (multiple answers possible): ‘No barriers’, ‘Lack of practice’, ‘Lack of knowledge’, ‘Lack of skills’, ‘Disbelief that it would change my confidence level’, ‘Feeling it would not be beneficial for me’, ‘Other, namely [text entry]’ | COM-B (capability, opportunity, motivation) | (12, 15, 26–28) |
| Opinions about improving SCI PA counseling |  |  |  |
| Think physical activity counseling is effective in improving exercise participation in adults with SCI | 7-point Likert scale (Agree-disagree) and ‘Unsure’ option | Self-efficacy | (29–31) |
| Want to improve my knowledge and skills | 7-point Likert scale (Agree-disagree) | COM-B (capability) | (15, 26) |
| Am interested to take part in a training/workshop on best practices | 7-point Likert scale (Agree-disagree) | COM-B (motivation) | (26) |
| Think there is value in taking part in a training/workshop on best practices | 7-point Likert scale (Agree-disagree) | COM-B (motivation) | (26) |
| Am motivated to take part in a training/workshop | 7-point Likert scale (Agree-disagree) | COM-B (motivation) | (26) |

**Table 4 Needs** Multiple choice and dichotomous (Yes/No) answer options.

| Construct | Options | Theory/approach | Reference |
| --- | --- | --- | --- |
| Needs and preferences using best practices for SCI PA counseling |  |  |  |
| Would use an online resource/toolkit outlining the evidence-based best practices | Yes/No |  | (12) |
| Would be interested in attending one or more online training session(s) or webinars about the new evidence-based best practices | Yes/No |  | (12) |
| Would be interested in online training modules that are free of charge to teach counselors the best practices | Yes/No |  | - |
| People that should be involved in the development of a training on SCI PA counseling | Multiple choice (multiple answers possible): ‘Person with SCI’, ‘Experienced counselor’, ‘Motivational Interviewing expert’, ‘SCI peer mentor’, ‘Researcher’, ‘Clinician’, ‘Panel members who co-created the best practices’, ‘Organization offering counseling support’, ‘Other, namely [text entry]’ | MI | (1, 12) |
| Aspects for in a training on SCI PA counseling | Multiple choice (multiple answers possible): ‘Short videos on the content’, ‘Example videos of a ‘good’ counseling session’, ‘Links to other resources/literature’, ‘Quizzes to test my knowledge’, ‘Discussion forum to post questions and share experiences with our counselors’, ‘Opportunities to receive feedback on my counseling skills’, ‘Practice/role play a counseling session with other participants’, ‘Practice/role play counseling sessions with actors’, ‘Other, namely [text entry]’ |  | (12, 27) |
| Topics related to SCI PA counseling | Multiple choice (multiple answers possible): ‘Physical activity barriers in adults with SCI’, ‘Assessing clients physical activity for adults with SCI’, ‘SCI Physical activity guidelines’, ‘Tailoring the support’, ‘Motivational Interviewing’, ‘Goal setting and action planning’, ‘Benefits of physical activity for adults with SCI’, ‘Building rapport with your client’, ‘Other, namely [text entry]’ | MI, behavior change techniques (goal setting, action planning), physical activity guidelines | (1, 12, 13, 15, 17, 19–25) |
| Types of resources or tools on SCI PA counseling | Multiple choice (multiple answers possible): ‘Action plan scheme’, ‘List of behavior change techniques’, ‘Example counseling question/flow chart’, ‘Videos with example counseling sessions’, ‘Other, namely [text entry]’ | Behavior change techniques (action planning) | (12, 19, 20) |

**Supplementary D. Results with incomplete cases included**

This supplement shows the tables of the results with incomplete cases included. The amount of respondents included (n) is shown in each table. At some point in the survey the remaining questions were only answered by people that completed the whole survey (complete cases). Those remaining questions were not answered by people that only filled in part of the survey (incomplete cases), therefore those results are not shown.

No notable differences were found between the results with the complete cases and the results with incomplete cases included. People with various types of expertise were not part of the incomplete cases (Table 1). Of those people, some Dutch respondents did not counsel people with SCI in an average work year. They did have experience in providing PA counseling. No notable differences were found in their counseling training.

**Table 1 Study population and expertise/background** characteristics of Canadian and Dutch respondents

Canada The Netherlands p-value n

Mean age in years ± SD (range) 34 ± 12 (21-62) 39 ± 11 (24-69) 0.09 CA 49, NL 48

Background and expertise* (%): CA 49, NL 48

Exercise or lifestyle counselor 14 (28.6) 25 (52.1) **0.02**

Therapeutic recreation therapist 21 (42.9) 3 (6.3) **<0.005**

SCI peer mentor 7 (14.3) 2 (4.2) 0.16

Physiotherapist 6 (12.2) 12 (25.0) 0.11

Occupational therapist 4 (8.2) 6 (12.5) 0.52

Other 19 (38.8) 12 (25.0)

How many adults with SCI do you counsel per year? (%) **<0.005** CA 49, NL 48

0 2 (4.1) 13 (27.1)

1-4 28 (57.1) 17 (35.4)

5-9 3 (6.1) 4 (8.3)

10-14 5 (10.2) 0 (0)

15-24 3 (6.1) 3 (6.3)

>25 8 (16.3) 11 (22.9)

How many years of experience do you have in providing physical

activity counseling? 1.00 CA 49, NL 48

None 2 (4.1) 3 (6.3)

Less than 1 year 8 (16.3) 8 (16.7)

Between 1-3 years 13 (26.5) 12 (25.0)

More than 3 years 26 (53.1) 25 (52.1)

Has received a formal training or workshop in Motivational

Interviewing 25 (51.0) 35 (72.9) **0.03** CA 49, NL 48

Has received other formal training or workshops in behavior

change or counseling techniques 33 (67.3) 20 (41.7) **0.01** CA 49, NL 48

*Notes*: CA = Canadian respondents, NL = Dutch respondents. *Respondents could select multiple background/expertise categories they belong in, therefore the p-value for the difference between Canadian and Dutch respondents is calculated for each category. No p-value is calculated for the category ‘other’ because groups that fall under this category differ.

No notable differences were found in counselors’ knowledge, skills and confidence levels using the best practices for SCI PA counseling between the results of the complete cases and results with incomplete cases included (Table 2).

**Table 2 Knowledge, skills and confidence levels** to use the best practices for SCI PA counseling

Canada The Netherlands n

**Best practices on *how* to have the conversation**

I have the knowledge and skills to build rapport and establish a relationship with my client with SCI CA 49, NL 45

Mode 6 6

Median (IQR) 6 (1) 6 (1)

Mean ± SD (range) 6 ± 1.1 (1-7) 6.2 ± 0.8 (4-7)

Number of unsure (%) 0 0

I am confident in my ability to build rapport and establish a relationship with my client with SCI CA 49, NL 45

Mode 6 6

Median (IQR) 6 (1) 6 (1)

Mean ± SD (range) 6.2 ± 0.7 (5-7) 5.9 ± 1.1 (4-7)

Number of unsure (%) 1 (2.0) 1

I have the knowledge and skills to use a client-centered approach following the spirit of Motivational Interviewing CA 49, NL 45

Mode 6 6

Median (IQR) 6 (1) 6 (0.8)

Mean ± SD (range) 5.4 ± 1.1 (2-7) 5.9 ± 1.1 (3-7)

Number of unsure (%) 2 (4.1) 1

I am confident in my ability to use a client-centered approach following the spirit of Motivational Interviewing CA 49, NL 45

Mode 6 6

Median (IQR) 6 (1) 6 (0)

Mean ± SD (range) 5.4 ± 1.4 (1-7) 5.9 ± 0.9 (2-7)

Number of unsure (%) 1 (2.0) 4

I have the knowledge and skills to tailor the support to my client’s motivation for physical activity and their current needs, values, wishes and preferences CA 49, NL 44

Mode 6 6

Median (IQR) 6 (1) 6 (1)

Mean ± SD (range) 5.7 ± 1.1 (1-7) 5.7 ± 1.1 (2-7)

Number of unsure (%) 0 1

I am confident in my ability to tailor the support to my client’s motivation for physical activity and their current needs, values, wishes and preferences CA 49, NL 44

Mode 6 6

Median (IQR) 6 (1) 6 (1)

Mean ± SD (range) 5.6 ± 1.1 (1-7) 5.7 ± 0.9 (2-7)

Number of unsure (%) 0 1

**Best practices on *what* to discuss**

I have the knowledge and skills to assess my client’s current physical activity behavior CA 49, NL 44

Mode 6 6

Median (IQR) 6 (1) 6 (1)

Mean ± SD (range) 5.5 ± 1.1 (1-7) 6.2 ± 0.8 (4-7)

Number of unsure (%) 0 1

I am confident in my ability to assess my client’s current physical activity behavior CA 49, NL 44

Mode 6 6

Median (IQR) 6 (1) 6 (1)

Mean ± SD (range) 5.3 ± 1.2 (1-7) 6.3 ± 0.7 (3-7)

Number of unsure (%) 2 (4.1) 1

I have the knowledge and skills to understand my client’s physical activity capability, opportunity and motivation CA 45, NL 42

Mode 6 6

Median (IQR) 6 (1) 6 (1)

Mean ± SD (range) 5.5 ± 1.1 (1-7) 5.6 ± 1.0 (2-7)

Number of unsure (%) 0 1

I am confident in my ability to understand my client’s physical activity capability, opportunity and motivation CA 46, NL 42

Mode 6 6

Median (IQR) 6 (1) 6 (1)

Mean ± SD (range) 5.3 ± 1.2 (2-7) 5.7 ± 1.0 (2-7)

Number of unsure (%) 1 (2.2) 1

I have the knowledge and skills to identify and understand physical activity barriers of my client with SCI CA 46, NL 42

Mode 6 6

Median (IQR) 6 (0.3) 6 (1)

Mean ± SD (range) 5.8 ± 1.0 (1-7) 5.6 ± 1.0 (2-7)

Number of unsure (%) 0 1

I am confident in my ability to identify and understand physical activity barriers of my client with SCI CA 46, NL 42

Mode 6 6

Median (IQR) 6 (1) 6 (0.5)

Mean ± SD (range) 5.7 ± 1.1 (1-7) 5.7 ± 0.9 (2-7)

Number of unsure (%) 1 (2.2) 1

I have the knowledge and skills to work together with my client with SCI to develop possible solutions to overcome their physical activity barriers CA 46, NL 42

Mode 6 6

Median (IQR) 6 (1) 6 (1)

Mean ± SD (range) 5.6 ± 1.1 (1-7) 5.5 ± 1.1 (2-7)

Number of unsure (%) 0 1

I am confident in my ability to together with my client with SCI to develop possible solutions to overcome their physical activity

barriers CA 46, NL 42

Mode 6 6

Median (IQR) 6 (1) 6 (1)

Mean ± SD (range) 5.5 ± 1.2 (1-7) 5.5 ± 1.1 (2-7)

Number of unsure (%) 1 (2.2) 1

I have the knowledge and skills to work together with my client with SCI to set a physical activity goal and create an action

plan CA 46, NL 41

Mode 6 6

Median (IQR) 6 (1) 6 (2)

Mean ± SD (range) 5.7 ± 1.0 (1-7) 5.3 ± 1.4 (2-7)

Number of unsure (%) 0 2

I am confident in my ability to work together with my client with SCI to set a physical activity goal and create an action

plan CA 46, NL 41

Mode 6 6

Median (IQR) 6 (1) 6 (1)

Mean ± SD (range) 5.7 ± 1.1 (1-7) 5.4 ± 1.2 (2-7)

Number of unsure (%) 0 2

I have the knowledge and skills to provide information to my client on benefits of physical activity for adults with SCI CA 46, NL 41

Mode 6 6

Median (IQR) 6 (1) 6 (1.5)

Mean ± SD (range) 5.7 ± 1.0 (2-7) 5.6 ± 1.6 (2-7)

Number of unsure (%) 0 1

I am confident in my ability to provide information to my client on benefits of physical activity for adults with SCI CA 46, NL 41

Mode 6 6

Median (IQR) 6 (1) 6 (0.8)

Mean ± SD (range) 5.5 ± 1.2 (2-7) 5.8 ± 1.3 (2-7)

Number of unsure (%) 0 1

I have the knowledge and skills to share the SCI-specific physical activity guidelines with my client with SCI CA 46, NL 41

Mode 6 5*

Median (IQR) 5 (2.5) 5 (2.8)

Mean ± SD (range) 5.0 ± 1.7 (1-7) 4.7 ± 1.9 (1-7)

Number of unsure (%) 1 (2.2) 1

I am confident in my ability to share the SCI-specific physical activity guidelines with my client with SCI CA 46, NL 41

Mode 6 6

Median (IQR) 6 (2) 6 (1.8)

Mean ± SD (range) 5.1 ± 1.6 (1-7) 5.2 ± 1.6 (1-7)

Number of unsure (%) 1 (2.2) 1

I have the knowledge and skills to share physical activity examples with my client with SCI CA 46, NL 41

Mode 6 6

Median (IQR) 6 (1) 6 (1.8)

Mean ± SD (range) 5.5 ± 1.2 (1-7) 5.2 ± 1.7 (1-7)

Number of unsure (%) 0 1

I am confident in my ability to share physical activity examples with my client with SCI CA 46, NL 41

Mode 6 6

Median (IQR) 6 (1) 6 (1)

Mean ± SD (range) 5.4 ± 1.3 (1-7) 5.4 ± 1.6 (1-7)

Number of unsure (%) 0 1

*Notes:* CA = Canadian respondents, NL = Dutch respondents. These questions were measured on a 7-point Likert scale (1= Strongly disagree, 2= Disagree, 3= Somewhat disagree, 4= Neither agree nor disagree, 5= Somewhat agree, 6= Agree, 7= Strongly agree). *There were multiple modes, the smallest value is shown.

Furthermore, there were no notable differences in barriers and the need on training in the best practices (Table 3+4). However, there was only one ‘incomplete case’ to include in each country for these variables.

**Table 3 Barriers and opinions** on improving physical activity counseling for adults with SCI

Canada The Netherlands p-value n

Are you experiencing any barriers to improving your knowledge

and skills on SCI-specific physical activity counseling?* (%) CA 46, NL 42

No, I do not experience any barriers 2 (4.3) 8 (19.0) **0.04**

Lack of time 34 (73.9) 22 (52.4) **0.04**

Lack of reimbursement 22 (47.8) 8 (19.0) **<0.005**

Lack of resources 11 (23.9) 4 (9.5) 0.07

Lack of interest 2 (4.3) 3 (7.1) 0.67

Feeling it would not be beneficial to me 2 (4.3) 1 (2.4) 1.00

Disbelief that it will change my counseling
knowledge/skills 0 (0) 1 (2.4) 0.48

Other 9 (19.6) 13 (31.0)

Are you experiencing any barriers to improving your confidence

in delivering physical activity counseling to adults with SCI?* (%) CA 46, NL 42

No, I do not experience any barriers 8 (17.4) 18 (42.9) **0.01**

Lack of practice 28 (60.7) 14 (33.3) **0.01**

Lack of knowledge 24 (52.2) 13 (31.0) 0.04

Lack of skills 13 (28.3) 7 (16.7) 0.20

Feeling it would not be beneficial to me 1 (2.2) 1 (2.4) 1.00

Disbelief that it will change my confidence level 1 (2.2) 1 (2.4) 1.00

Other 5 (10.7) 3 (7.1)

I want to improve my knowledge and skills on SCI-specific

physical activity counseling°

Mean ± SD (range) 6.3 ± 0.7 (5-7) 5.1 ± 1.5 (1-7) **<0.005** CA 46, NL 42

Would use an online resource/toolkit outlining the evidence-based

best practices (%) 46 (100.0) 35 (83.3) **<0.005** CA 46, NL 42

*Notes*: CA= Canadian respondents, NL=Dutch respondents. *Respondents could select multiple barriers, therefore the p-value for the difference between Canadian and Dutch respondents is calculated for each category. No p-value is calculated for the category ‘other’ because groups that fell under this category differ. °Measured on a 7-point Likert scale (1=strongly disagree, 2=disagree, 3=somewhat disagree, 4=neither agree nor disagree, 5=somewhat agree, 6=agree, 7=strongly agree).

**Supplementary E. Results**

This supplementary file contains the results of some additional questions on demographic information, sexual orientation and ethnicity (Table 1). Most of the respondents were straight (Canada 91%; Netherlands 78%) and of European descent (white, Canada 80%; Netherlands ±93%). Furthermore, the mode, median (IQR) and mean (SD) are shown of counselors’ knowledge, skills and confidence levels to use the best practices for SCI PA counseling (Table 2). Table 3 shows the mean difference in knowledge, skills and confidence of counselors’ to use the best practices of Canadian counselors compared to Dutch counselors.

**Table 1 Study population** demographic characteristics of Canadian and Dutch respondents

Canada The Netherlands p-value
 (n=45) (n=41)

Sexual orientation (%): 0.09

Straight 41 (91.1) 32 (78.0)

Bisexual 1 (2.2) 0 (0)

Lesbian 0 (0) 1 (2.4)

Asexual 0 (0) 0 (0)

Gay 0 (0) 0 (0)

Pansexual 0 (0) 0 (0)

Another sexual orientation 0 (0) 0 (0)

Prefer not to answer 3 (6.7) 8 (19.5)

Ethnicity (%)*:

White 36 (80.0)

Latino 2 (4.4)

Native 2 (4.4)

South Asian 2 (4.4)

Black 1 (2.2)

East/Southeast Asian 1 (2.2)

Middle-Eastern 0 (0)

Another race category 0 (0)

I do not know 0 (0)

Prefer not to answer 4 (8.9)

In which country were you born? (%)*

The Netherlands 38 (92.7)

Other (Greece, Indonesia, Austria) 3 (7.3)

In which country was your mother born? (%)*

The Netherlands 38 (92.7)

Other (Germany, Indonesia) 2 (4.9)

Missing 1 (2.4)

In which country was your father born? (%)*

The Netherlands 37 (90.2)

Other (Greece, Indonesia) 3 (7.3)

Missing 1 (2.4)

*Notes*: *Because of differences in measuring ethnic descent between the countries, significance of the difference between ethnic descent of the Canadian and Dutch respondents could not be calculated.

**Table 2 Knowledge, skills and confidence levels** to use the best practices for SCI PA counseling

Canada The Netherlands (n=45) (n=41)

**Best practices on *how* to have the conversation**

I have the knowledge and skills to build rapport and establish a relationship with my client with SCI

Mode 6 6

Median (IQR) 6 (1) 6 (1)

Mean ± SD (range) 6.0 ± 0.9 (3-7) 6.3 ± 0.7 (4-7)

Number of unsure (%) 0 (0) 0 (0)

I am confident in my ability to build rapport and establish a relationship with my client with SCI

Mode 6 6

Median (IQR) 6 (1) 6 (1)

Mean ± SD (range) 6.3 ± 0.7 (5-7) 6.5 ± 0.6 (5-7)

Number of unsure (%) 1 (2.2) 1 (2.4)

I have the knowledge and skills to use a client-centered approach following the spirit of Motivational Interviewing

Mode 6 6

Median (IQR) 6 (1) 6 (1)

Mean ± SD (range) 5.4 ± 1.1 (2-7) 5.9 ± 1.1 (3-7)

Number of unsure (%) 2 (4.4) 1 (2.4)

I am confident in my ability to use a client-centered approach following the spirit of Motivational Interviewing

Mode 6 6

Median (IQR) 6 (1) 6 (0)

Mean ± SD (range) 5.3 ± 1.4 (1-7) 6.0 ± 0.9 (2-7)

Number of unsure (%) 1 (2.2) 3 (7.3)

I have the knowledge and skills to tailor the support to my client’s motivation for physical activity and their current needs, values, wishes and preferences

Mode 6 6

Median (IQR) 6 (1) 6 (0.8)

Mean ± SD (range) 5.7 ± 1.2 (1-7) 5.8 ± 0.9 (3-7)

Number of unsure (%) 0 (0) 1 (2.4)

I am confident in my ability to tailor the support to my client’s motivation for physical activity and their current needs, values, wishes and preferences

Mode 6 6

Median (IQR) 6 (1) 6 (0)

Mean ± SD (range) 5.6 ± 1.1 (1-7) 5.9 ± 0.6 (4-7)

Number of unsure (%) 0 (0) 1 (2.4)

**Best practices on *what* to discuss**

I have the knowledge and skills to assess my client’s current physical activity behavior

Mode 6 6

Median (IQR) 6 (1) 6 (1)

Mean ± SD (range) 5.5 ± 1.1 (1-7) 6.2 ± 0.8 (4-7)

Number of unsure (%) 0 (0) 1 (2.4)

I am confident in my ability to assess my client’s current physical activity behavior

Mode 6 6

Median (IQR) 6 (1) 6 (1)

Mean ± SD (range) 5.4 ± 1.2 (1-7) 6.3 ± 0.7 (5-7)

Number of unsure (%) 2 (4.4) 1 (2.4)

I have the knowledge and skills to understand my client’s physical activity capability, opportunity and motivation

Mode 6 6

Median (IQR) 6 (1) 6 (1)

Mean ± SD (range) 5.5 ± 1.1 (1-7) 5.6 ± 1.1 (2-7)

Number of unsure (%) 1 (2.2) 1 (2.4)

I am confident in my ability to understand my client’s physical activity capability, opportunity and motivation

Mode 6 6

Median (IQR) 6 (1) 6 (1)

Mean ± SD (range) 5.3 ± 1.2 (2-7) 5.7 ± 1.0 (2-7)

Number of unsure (%) 1 (2.2) 1 (2.4)

I have the knowledge and skills to identify and understand physical activity barriers of my client with SCI

Mode 6 6

Median (IQR) 6 (0) 6 (1)

Mean ± SD (range) 5.8 ± 1.1 (1-7) 5.6 ± 1.0 (2-7)

Number of unsure (%) 0 (0) 1 (2.4)

I am confident in my ability to identify and understand physical activity barriers of my client with SCI

Mode 6 6

Median (IQR) 6 (1) 6 (0.8)

Mean ± SD (range) 5.7 ± 1.1 (1-7) 5.7 ± 1.0 (2-7)

Number of unsure (%) 1 (2.2) 1 (2.4)

I have the knowledge and skills to work together with my client with SCI to develop possible solutions to overcome their physical activity barriers

Mode 6 6

Median (IQR) 6 (1) 6 (1)

Mean ± SD (range) 5.6 ± 1.2 (1-7) 5.5 ± 1.1 (2-7)

Number of unsure (%) 0 (0) 1 (2.4)

I am confident in my ability to together with my client with SCI to develop possible solutions to overcome their physical activity barriers

Mode 6 6

Median (IQR) 6 (1) 6 (1)

Mean ± SD (range) 5.5 ± 1.2 (1-7) 5.5 ± 1.1 (2-7)

Number of unsure (%) 1 (2.2) 1 (2.4)

I have the knowledge and skills to work together with my client with SCI to set a physical activity goal and create an action plan

Mode 6 6

Median (IQR) 6 (1) 6 (2)

Mean ± SD (range) 5.7 ± 1.0 (1-7) 5.3 ± 1.4 (2-7)

Number of unsure (%) 0 (0) 3 (7.3)

I am confident in my ability to work together with my client with SCI to set a physical activity goal and create an action plan

Mode 6 6

Median (IQR) 6 (1) 6 (1)

Mean ± SD (range) 5.7 ± 1.1 (1-7) 5.4 ± 1.3 (2-7)

Number of unsure (%) 0 (0) 3 (7.3)

I have the knowledge and skills to provide information to my client on benefits of physical activity for adults with SCI

Mode 6 6

Median (IQR) 6 (1) 6 (1)

Mean ± SD (range) 5.7 ± 1.0 (2-7) 5.7 ± 1.5 (2-7)

Number of unsure (%) 0 (0) 2 (4.9)

I am confident in my ability to provide information to my client on benefits of physical activity for adults with SCI

Mode 6 6

Median (IQR) 6 (1) 6 (1)

Mean ± SD (range) 5.5 ± 1.2 (2-7) 5.9 ± 1.2 (2-7)

Number of unsure (%) 0 (0) 2 (4.9)

I have the knowledge and skills to share the SCI-specific physical activity guidelines with my client with SCI

Mode 6 5*

Median (IQR) 5.5 (2) 5 (2)

Mean ± SD (range) 5.0 ± 1.6 (1-7) 4.7 ± 1.8 (1-7)

Number of unsure (%) 1 (2.2) 2 (4.9)

I am confident in my ability to share the SCI-specific physical activity guidelines with my client with SCI

Mode 6 6

Median (IQR) 5.5 (2) 6 (1)

Mean ± SD (range) 5.0 ± 1.6 (1-7) 5.3 ± 1.6 (1-7)

Number of unsure (%) 1 (2.2) 2 (4.9)

I have the knowledge and skills to share physical activity examples with my client with SCI

Mode 6 6

Median (IQR) 6 (1) 6 (1)

Mean ± SD (range) 5.5 ± 1.3 (1-7) 5.3 ± 1.7 (1-7)

Number of unsure (%) 0 (0) 2 (4.9)

I am confident in my ability to share physical activity examples with my client with SCI

Mode 6 6

Median (IQR) 6 (1) 6 (1)

Mean ± SD (range) 5.4 ± 1.4 (1-7) 5.4 ± 1.6 (1-7)

Number of unsure (%) 0 (0) 2 (4.9)

*Notes:* These questions were measured on a 7-point Likert scale (1= Strongly disagree, 2= Disagree, 3= Somewhat disagree, 4= Neither agree nor disagree, 5= Somewhat agree, 6= Agree, 7= Strongly agree). *There were multiple modes, the smallest value is shown.

**Table 3a Linear regression analysis** Canada compared with the Netherlands in knowledge, skills and confidence to use the best practices.

Mean difference 95% confidence interval p-value

**Best practices on *how* to have the conversation**

Knowledge and skills 0.79 -0.28 – 1.86 0.15

Confidence 0.94 0.01 – 1.86 **0.05**

**Best practices on *what* to discuss**

Knowledge and skills -0.97 -4.13 – 2.19 0.54

Confidence 1.35 -1.81 – 4.50 0.40

*Notes*: unsure was coded as 4=neither agree nor disagree. Canada was compared with the Netherlands. See Supplement E Table 3b for unsure coded as 2=disagree.

**Table 3b Linear regression analysis** Canada compared with the Netherlands in knowledge, skills and confidence to use the best practices.

Mean difference 95% confidence interval p-value

**Best practices on *how* to have the conversation**

Knowledge and skills 0.78 -0.37 – 1.93 0.18

Confidence 0.78 -0.32 – 1.88 0.16

**Best practices on *what* to discuss**

Knowledge and skills -1.52 -4.97 – 1.94 0.39

Confidence 0.98 -2.54 – 4.50 0.58

*Notes*: unsure was coded as 2=disagree. Canada was compared with the Netherlands. See Supplement E Table 3a for unsure coded as 4=neither agree nor disagree.

**References**

1. Hoekstra F, Gainforth HL, Broeksteeg R, Corras S, Collins D, Gaudet S, Giroux EE, McCallum S, Ma JK, Rakiecki D, et al. Theory- and evidence-based best practices for physical activity counseling for adults with spinal cord injury. J. Spinal Cord Med. 2023 Mar.

2. SCI Action Canada Lab [Internet]. Kelowna; University of British Columbia. [cited 2022 Feb 14]; [about 1 screen]. Available from: https://sciactioncanada.ok.ubc.ca/

3. ReSpAct [Internet]. Groningen [cited 2022 Feb 21]; [about 2 screens]. Available from: http://www.respact.nl/

4. Stichting Special Heroes Nederland [Internet]. Arnhem: Stichting Special Heroes Nederland [cited 2022 Feb 21]; [about 2 screens]. Available from: https://specialheroes.nl/

5. Giroux E. SCI CBO - Updated List [Excel data file]. Giroux E; 2022 [cited 2022 Jul 28].

6. Statistics Canada [Internet]. Statistics Canada. Provinces and territories. 2021 Feb 15 [cited 2022 Jul 28]; [about 1 screen]. Available from: https://www.statcan.gc.ca/en/reference/province

7. Fraser G. Evaluating inclusive gender identity measures for use in quantitative psychological research. Psychol Sex 2018 Jul; 9(4).

8. Vanderbilt University [Internet]. Nashville: Vanderbilt University; c2022. How to Ask About Sexuality/Gender. [cited 2022 Jul 22]; [about 6 screens]. Available from: https://www.vanderbilt.edu/lgbtqi/resources/how-to-ask-about-sexuality-gender

9. Statistics Canada [Internet]. Statistics Canada. Ethnic and cultural origins of Canadians: Portrait of a rich heritage. 2017 Oct 25 [cited 2022 Jul 22]; [about 5 screens]. Available from: https://www12.statcan.gc.ca/census-recensement/2016/as-sa/98-200-x/2016016/98-200-x2016016-eng.cfm

10. Banner D, Bains M, Carroll S, Kandola DK, Rolfe DE, Wong C. Patient and Public Engagement in Integrated Knowledge Translation Research: Are we there yet? Res Involv Engagem 2019 Feb; 5.

11. MINT [Internet]. Motivational Interviewing Network of Trainers; c2021. Understanding Motivational Interviewing. [cited 2022 Jul 29]; [about 3 screens]. Available from: https://motivationalinterviewing.org/understanding-motivational-interviewing

12. Ma JK, Cheifetz O, Todd KR, Chebaro C, Phang SH, Shaw RB. Co-development of a physiotherapist-delivered physical activity intervention for adults with spinal cord injury. Spinal Cord 2020 Jul; 58(7): 778-786.

13. Martin Ginis KA, Scheer JW van der, Latimer-Cheung AE, Barrow A, Bourne C, Carruthers P, et al. Evidence-based scientific exercise guidelines for adults with spinal cord injury: an update and a new guideline. Spinal Cord 2018 Apr; 56(4): 308–21.

14. World Health Organization [Internet]. World Health Organization; c2022. Physical activity; 2020 Nov 26 [cited 2022 Mar 15]; [about 8 screens]. Available from: https://www.who.int/news-room/fact-sheets/detail/physical-activity

15. Social Change UK. A guide on The COM-B Model of Behaviour. London: Social Change UK; 2019.

16. Fekete C, Rauch A. Correlates and determinants of physical activity in persons with spinal cord injury: A review using the International Classification of Functioning, Disability and Health as reference framework. Disabil Health J 2012; 5: 140-150.

17. Williams TL, Smith B, Papathomas A. The barriers, benefits and facilitators of leisure time physical activity among people with spinal cord injury: a meta-synthesis of qualitative findings. Health Psychol Rev 2014 Feb; 8(4): 404-425.

18. Lawrason SVC, Todd KR, Shaw RB, Martin Ginis KA. Physical activity among individuals with spinal cord injury who ambulate: a systematic scoping review. Spinal Cord 2020 Jul; 58(7): 735-745.

19. Arbour-Nicitopoulos KP, Martin Ginis KA, Latimer AE. Planning, Leisure-Time Physical Activity, and Coping Self-Efficacy in Persons With Spinal Cord Injury: A Randomized Controlled Trial. Arch Phys Med Rehabil 2009 Dec; 90: 2003-2011.

20. Latimer AE, Martin Ginis KA, Arbour KP. The efficacy of an implementation intention intervention for promoting physical activity among individuals with spinal cord injury: A randomized controlled trial. Rehabil Psychol 2006; 51(4): 273-280.

21. Tomasone JR, Wesch NN, Martin Ginis KA, Noreau L. Spinal Cord Injury, Physical Activity, and Quality of Life: A Systematic Review. Kinesiol Rev. 2013 May; 2: 113-129.

22. Scheer JW van der, Martin Ginis KA, Ditor DS, Goosey-Tolfrey Vl, Hicks AL, West CR, et al. Effects of exercise on fitness and health of adults with spinal cord injury. Neurology 2017 Aug; 89: 736-745.

23. Hoekstra F, McBride CB, Borisoff J, Fetterly M, Ginis S, Latimer-Cheung AE, Ma JK, Maffin J, Mah L, West CR, et al. Translating the international scientific spinal cord injury exercise guidelines into community and clinical practice guidelines: a Canadian evidence-informed resource. Spinal Cord 2020; 58: 647-657.

24. Martin Ginis KA, Arbour-Nicitopoulos KP, Latimer AE, Buchholz AC, Bray SR, Craven BC, Hayes KC, Hicks AL, McColl MA, Potter PJ, et al. Leisure time physical activity in a population-based sample of people with spinal cord injury part II: activity types, intensities, and durations. Arch Phys Med Rehabil 2010; 91(5): 729-733.

25. Perrier MJ, Stork MJ, Martin Ginis KA. Type, intensity and duration of daily physical activities performed by adults with spinal cord injury. Spinal Cord 2017; 55: 64-70.

26. West R, Michie S. A brief introduction to the COM-B Model of behaviour and the PRIME Theory of motivation. Qeios 2020 Apr.

27. Shirley D, Ploeg HP van der, Bauman AE. Physical Activity Promotion in the Physical Therapy Setting: Perspectives From Practitioners and Students. Phys Ther 2010 Sep; 90(9): 1311-1322.

28. Schwarzer R, Lippke S, Luszczynska A. Mechanisms of health behavior change in persons with chronic illness or disability: the Health Action Process Approach (HAPA). Rehabil Psychol 2011 Aug; 56(3): 161-170.

29. Barnes KL. Applying Self-Efficacy Theory to Counselor Training and Supervision: A Comparison of Two Approaches. CES 2004; 44(1): 56-69.

30. Larson LM, Daniels JA. Review of the Counseling Self-Efficacy Literature. Couns Psychol 1998; 26(2): 179–218.

31. Hahn D, Weck F, Witthöft M, Kühne F. Assessment of Counseling Self-Efficacy: Validation of the German Counselor Activity Self-Efficacy Scales-Revised. Front Psychol 2021 Dec; 12: 5832.
